# Exocrine pancreas proteases regulate β-cell proliferation in zebrafish ciliopathy models and in murine systems

**DOI:** 10.1242/bio.046839

**Published:** 2021-06-14

**Authors:** Timothy L. Hostelley, Jessica E. Nesmith, Emily Larkin, Amanda Jones, Daniel Boyes, Carmen C. Leitch, Magali Fontaine, Norann A. Zaghloul

**Affiliations:** 1 Division of Endocrinology, Diabetes and Nutrition, Department of Medicine, University of Maryland School of Medicine, Baltimore, MD, 21201, USA; 2 Department of Pathology, University of Maryland School of Medicine, Baltimore, MD, 21201, USA; 3 Program in Personalized and Genomic Medicine, University of Maryland School of Medicine, Baltimore, MD, 21201, USA

**Keywords:** β-cells, Ciliopathies, Diabetes, Zebrafish

## Abstract

Pancreatic β-cells are a critical cell type in the pathology of diabetes. Models of genetic syndromes featuring diabetes can provide novel mechanistic insights into regulation of β-cells in the context of disease. We previously examined β-cell mass in models of two ciliopathies, Alström Syndrome (AS) and Bardet-Biedl Syndrome (BBS), which are similar in the presence of metabolic phenotypes, including obesity, but exhibit strikingly different rates of diabetes. Zebrafish models of these disorders show deficient β-cells with diabetes in AS models and an increased β-cells absent diabetes in BBS models, indicating β-cell generation or maintenance that correlates with disease prevalence. Using transcriptome analyses, differential expression of several exocrine pancreas proteases with directionality that was consistent with β-cell numbers were identified. Based on these lines of evidence, we hypothesized that pancreatic proteases directly impact β-cells. In the present study, we examined this possibility and found that pancreatic protease genes contribute to proper maintenance of normal β-cell numbers, proliferation in larval zebrafish, and regulation of AS and BBS β-cell phenotypes. Our data suggest that these proteins can be taken up directly by cultured β-cells and *ex vivo* murine islets, inducing proliferation in both. Endogenous uptake of pancreatic proteases by β-cells was confirmed *in vivo* using transgenic zebrafish and in intact murine pancreata. Taken together, these findings support a novel proliferative signaling role for exocrine pancreas proteases through interaction with endocrine β-cells.

## Abstract (precis)

**Summary:** In this paper, we examine the role of exocrine pancreas protease genes in β-cell number using zebrafish and murine models, and identify a novel relationship between the two.

## INTRODUCTION

With the increase in global rates of obesity, the prevalence of type 2 diabetes mellitus (T2DM) has also risen dramatically. Despite this, the mechanisms mediating differential susceptibility to T2DM in the presence of obesity are not well understood. Rare genetic syndromes characterized by the presence of obesity with and without T2DM can provide important mechanistic insight into genetic susceptibility to T2DM. The ciliopathies are a group of genetic disorders that can be informative because the two that are uniquely characterized by highly penetrant and early-onset obesity, Alström Syndrome (AS) and Bardet Biedl Syndrome (BBS), exhibit markedly different rates of T2DM (Girard and Petrovsky, 2011; Lodh et al., 2014). AS is caused by mutations in a single gene, *alms1*, encoding the ALMS1 protein which localizes to the basal body of primary cilia (Girard and Petrovsky, 2011; Marshall et al., 2012). BBS is caused by mutations in any of 21 known BBS genes, which largely localize to the basal body and primary cilia (Aldahmesh et al., 2014; Forsythe and Beales, 2013). In previous studies, we explored β-cell phenotypes in zebrafish models of these two conditions and identified significantly decreased β-cell proliferation and numbers by either knockdown or knockout of *alms1* (Nesmith et al., 2019) and significantly increased β-cell proliferation and numbers with loss of *bbs1* or *bbs4*, two commonly mutated BBS causing genes (Lodh et al., 2015). These observations suggest that AS and BBS can provide novel insight into pathways regulating β-cell proliferation in the context of T2DM.

We recently reported whole transcriptome data of zebrafish models of AS and BBS using RNA sequencing (Hostelley et al., 2016). These data revealed significant expression differences in exocrine pancreatic protease genes correlating with β-cell loss in AS models and β-cell increase in BBS models. Exocrine pancreatic insufficiency accompanying diabetes mellitus has been reported in both types 1 and 2 diabetes. For example, changes in exocrine pancreas precede β-cell loss in type 1 diabetes (Canaway et al., 2000; Laass et al., 2004; Piciucchi et al., 2015). In addition, pancreatic enzymes can be detected in circulation and are reduced in T2DM (Bharmal et al., 2017; Yadav et al., 2013). However, a role for the exocrine pancreas in diabetes development is unknown.

Here, we set out to test the possibility that exocrine pancreas proteases can directly impact production of β-cells. Using zebrafish, cultured mouse β-cells, and *ex vivo* cultured mouse islets, we examined this possibility in both wild-type conditions and ciliopathy models carrying discrepant β-cell proliferation. We found that overexpression or loss of protease gene expression in zebrafish larvae resulted in increased and reduced β-cell numbers, respectively. These effects were consistent with our observations in cultured murine β-cells and *ex vivo* isolated islets in which we not only observed increased β-cell proliferation in the presence of exocrine protease proteins, but also uptake of these proteins. These observations suggest a previously unappreciated role for exocrine pancreatic enzyme proteins in regulating β-cell proliferation, a finding that may have direct relevance to human diabetes.

## RESULTS

### Exocrine pancreas proteases are significant contributors to β-cell production

**Fig. 1.**
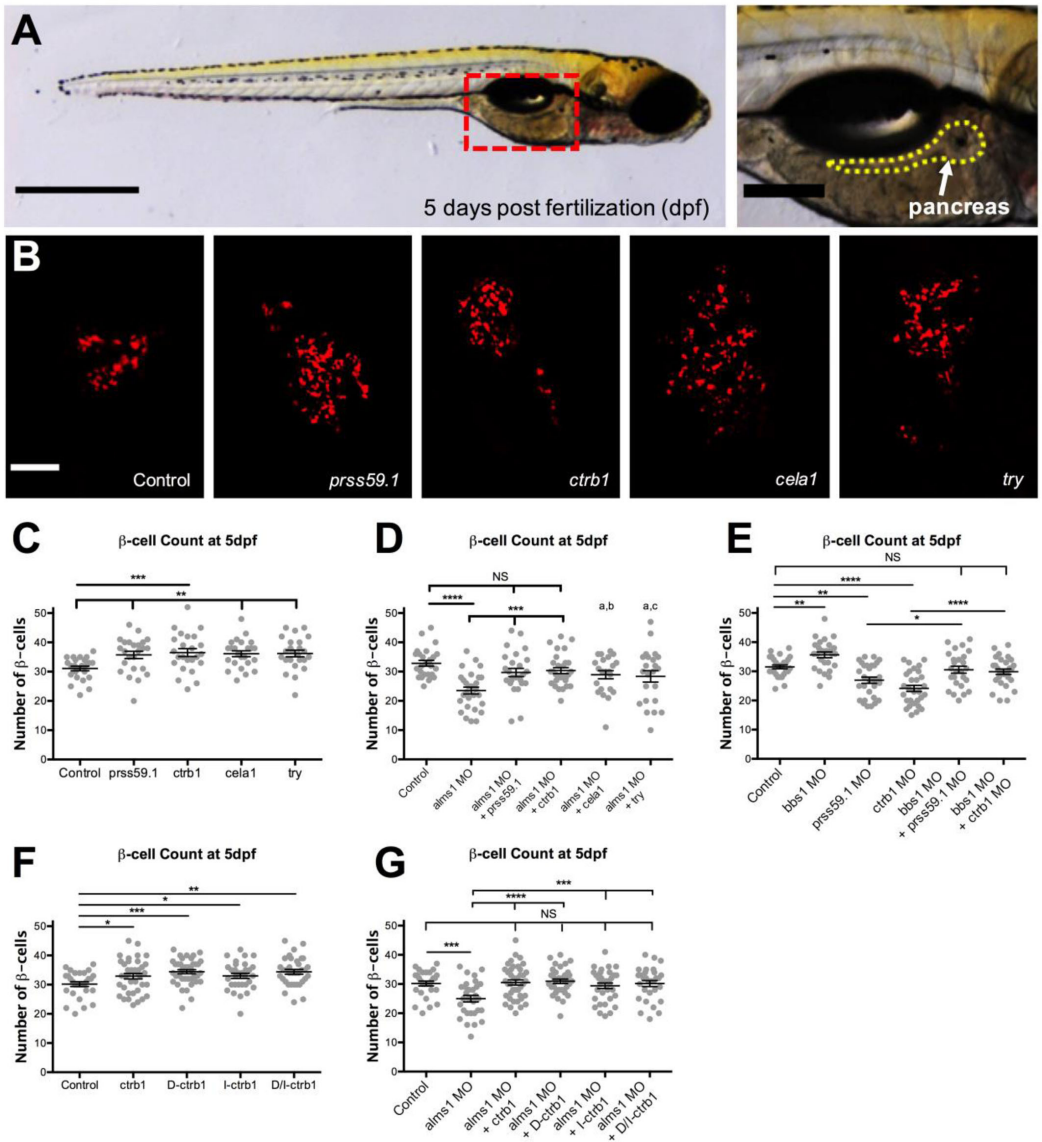
**Exocrine pancreas proteases contribute to β-cell production.** (A) Brightfield image of representative 5 dpf larva shown, boxed area indicates location of pancreas. Scale bars: 500 μm (left) and 100 μm (right). (B) Visualization of individual mCherry-expressing β-cells in control and protease mRNA injected Tg(*ins:mCherry*) larvae at 5 dpf. Scale bar: 10 μm. (C) β-cell count in control (*n*=24) and protease mRNA (*n*=24 for five each) injected animals at 5 dpf. (D) β-cell count in control (*n*=26), *alms1* MO (*n*=29), and *alms1* MO plus protease mRNA (*n*=26, 29, 22 and 23, respectively) injected larvae at 5 dpf. a=* compared to control, b=** compared to *alms1* MO, and c=* compared to *alms1* MO. (E) β-cell count in control (*n*=27), *bbs1* MO (*n*=26), protease MO (*n*=29 for both), and *bbs1* MO plus protease MO (*n*=26 and 29, respectively) injected larvae at 5 dpf. (F) β-cell count in control (*n*=29), *ctrb1* mRNA (*n*=38), catalytically dead (D)-*ctrb1* mRNA (*n*=40), inactivatable (I)-*ctrb1* mRNA (*n*=31), and both catalytically dead and inactivatable (D/I)-*ctrb1* mRNA (*n*=35) injected animals at 5 dpf. (G) β-cell count in control (*n*=29), *alms1* MO (*n*=30), and *alms1* MO plus: *ctrb1* mRNA (*n*=40), catalytically dead (D)-*ctrb1* mRNA (*n*=35), inactivatable (I)-*ctrb1* mRNA (*n*=33), or both catalytically dead and inactivatable (D/I)-*ctrb1* mRNA (*n*=30) injected larvae at 5 dpf. All statistics, Ordinary one-way ANOVA, error bars represent standard error of the mean, symbols represent the following significance: NS=p>0.05, *=*P*<0.05, **=*P*<0.01, ***=*P*<0.001, ****=*P*<0.0001.

To identify a role for exocrine pancreas proteases in regulating β-cells, we first overexpressed each protease in transgenic zebrafish embryos in which β-cells can be visualized by mCherry expression driven by the insulin promoter (Pisharath et al., 2007). We generated full length mRNA for each protease gene, *prss2 (prss59.1)*, *ctrb1*, *ela1 (cela1)* and *prss1 (try*) which encode Protease serine 2, Chymotrypsinogen B1, Chymotrypsin-like elastase family member 1 and Protease serine 1, respectively. The mRNA was injected into 1–2 cell stage Tg(*insa*:mCherry) zebrafish embryos and at 5 days post fertilization (dpf) – at which point there is a single principal endocrine islet present in a functional pancreas – the number of β-cells was quantified (Fig. 1A). In each case, protease gene overexpression resulted in a significant increase in the number of β-cells when compared to control animals (Fig. 1B). Control animals displayed an average of ∼31±0.78 β-cells (*n*=24), consistent with previous reports (Lodh et al., 2015; Maddison and Chen, 2012). In comparison, we found an average of ∼36 β-cells after *prss59.1* (±1.23, *P*=0.004, *n*=24), *cela1* (±0.99, *P*=0.0018, *n*=24) and *try* (±1.13, *P*=0.0015, *n*=24) overexpression and an average of ∼37 β-cells after *ctrb1* overexpression (±1.34, *P*=0.0008, *n*=24) (Fig. 1C). These results suggest that increased levels of exocrine pancreas proteases can increase β-cell production.

The relevance of protease expression changes in the ciliopathy models was verified using reintroduced protease expression in the AS model, normally exhibiting decreased expression (Hostelley et al., 2016). Endogenous *alms1* knockdown utilized a splice blocking morpholino (MO) previously validated to suppress protein production and recapitulating β-cell phenotypes in genomic knockout mutants (Lodh et al., 2015; Nesmith et al., 2019). We injected the MO in combination with full-length mRNA of individual proteases. At 5 dpf we found the previously reported reduction in β-cell number upon injection with *alms1* MO alone, [∼24±1.16 (*P*=<0.0001, *n*=29)] when compared to control MO injected animals (∼32±0.77, *n*=24; Fig. 1D). In contrast, *alms1* MO combined with overexpression of *prss59.1* (∼30±1.42, *P*=0.0008, *n*=26), *ctrb1* (∼30±1.02, *P*=0.0002, *n*=29), *cela1* (∼29±1.39, *P*=0.0051, *n*=22), or *try* (∼28±1.95, *P*=0.0108, *n*=23) significantly increased β-cell numbers when compared with *alms1* MO (Fig. 1D). Furthermore, β-cell numbers in *alms1* MO with either *prss59.1* or *ctrb1* RNA was not significantly different than control β-cell numbers (*P*=0.0919 and *P*=0.692), suggesting protease-induced rescue. We next sought to determine if protease gene expression levels are important for normal β-cell production. We used splice blocking MOs targeting either *prss59.1* or *ctrb1* transcripts, selecting genes that we previously found to be either upregulated in BBS models or downregulated in Alström models, respectively (Hostelley et al., 2016). After validating the efficacy of the MOs to suppress protease expression (Fig. S1A,B), we examined effects on β-cells. A significant reduction in β-cell number upon *prss59.1* MO (∼27±1.07, *P*=0.0014, *n*=29) or *ctrb1* MO (∼24±1.07, *P*=<0.0001, *n*=29) was observed (Fig. 1E). These results suggest that protease expression is important for normal β-cell production.

The BBS model exhibits both increased β-cells and increased protease expression, leading us to postulate protease knockdown could restore normal β-cell numbers (Hostelley et al., 2016; Lodh et al., 2015). A validated splice blocking MO targeting *bbs1* (Leitch et al., 2014) was injected with either *prss59.1* MO or *ctrb1* MO. *bbs1* MO increased the β-cell numbers (∼36±1.00, *P*=0.0047, *n*=26) when compared to control, which is consistent with previously reported findings (Fig. 1E; Lodh et al., 2015). Co-injection of MOs targeting either *prss59.1* or *ctrb1* returned β-cell numbers to control levels (*P*=0.4973 and *P*=0.2491), though they still increased compared to *prss59.1* MO (∼31±1.18, *P*=0.0126, *n*=26), or *ctrb1* MO (∼30±0.91 *n*=29; Fig. 1E). These results suggest an important role for exocrine pancreas protease expression in β-cell number.

### Inactive and catalytically dead ctrb1 mutants enhance β-cell production

The exocrine pancreas proteases are produced by acinar cells in the inactive or zymogen state and become activated by enterokinase cleavage in the small intestine (Whitcomb and Lowe, 2007). In light of this activation step, we questioned whether protease activity contributed to the observed β-cell effects. To test this, we focused on *ctrb1* since its mRNA produced the significant increase in β-cell number (Fig. 1D). We introduced mutations into *ctrb1* sequence that would render the resulting protein either inactivatable by cleavage (I-*ctrb1*), catalytically dead (D-*ctrb1*), or both inactivatable and catalytically dead (D/I-*ctrb1*; Fig. S2). Full-length mRNA of these *ctrb1* mutants were injected into Tg(*insa*:mCherry) zebrafish embryos and β-cell numbers were quantified at 5 dpf. We found overexpressing any *ctrb1* mutant yielded a significant increase in the number of β-cells (D-*ctrb1* ∼34±0.69, *P*=0.0009, *n*=40; I-*ctrb1* 33±0.87, *P*=0.0362, *n*=31; D/I*-ctrb1* ∼34±0.82, *P*=0.0013, *n*=35) when compared to control animals (∼30±0.82, *n*=29) (Fig. 1F), suggesting that chymotrypsinogen B1 zymogen increases normal β-cell production.

The ability of inactivatable and/or catalytically dead *ctrb1* to mimic the rescue of AS-induced β-cell decrease was tested by injecting full-length mutant *ctrb1* mRNA, alongside *alms1* MO, into Tg(*insa*:mCherry) zebrafish embryos. We found a significant increase in the number of β-cells at 5 dpf in mutant *ctrb1* mRNA-injected larvae in combination with the *alms1* MO (D-*ctrb1* ∼31±0.77, *P*=<0.0001, *n*=35; I-*ctrb1* ∼29±0.96, *P*=0.0008, *n*=33; D/I*-ctrb1* ∼30±1.03, *P*=0.0001, *n*=30) than with the *alms1* MO alone (∼25±1.12, *n*=30) (Fig. 1G). Furthermore, *ctrb1* mutant injection into the AS model rescued β-cell numbers to control levels (∼30±0.82, *n*=29). In total, these results demonstrate that protease activation by cleavage and catalytic activity are not required for *ctrb1* to rescue the loss of β-cells, in both control and in the AS model, supporting a role for the inactive proteases in driving the β-cell effect.

### Exocrine proteases specifically impact β-cell proliferation

**Fig. 2.**
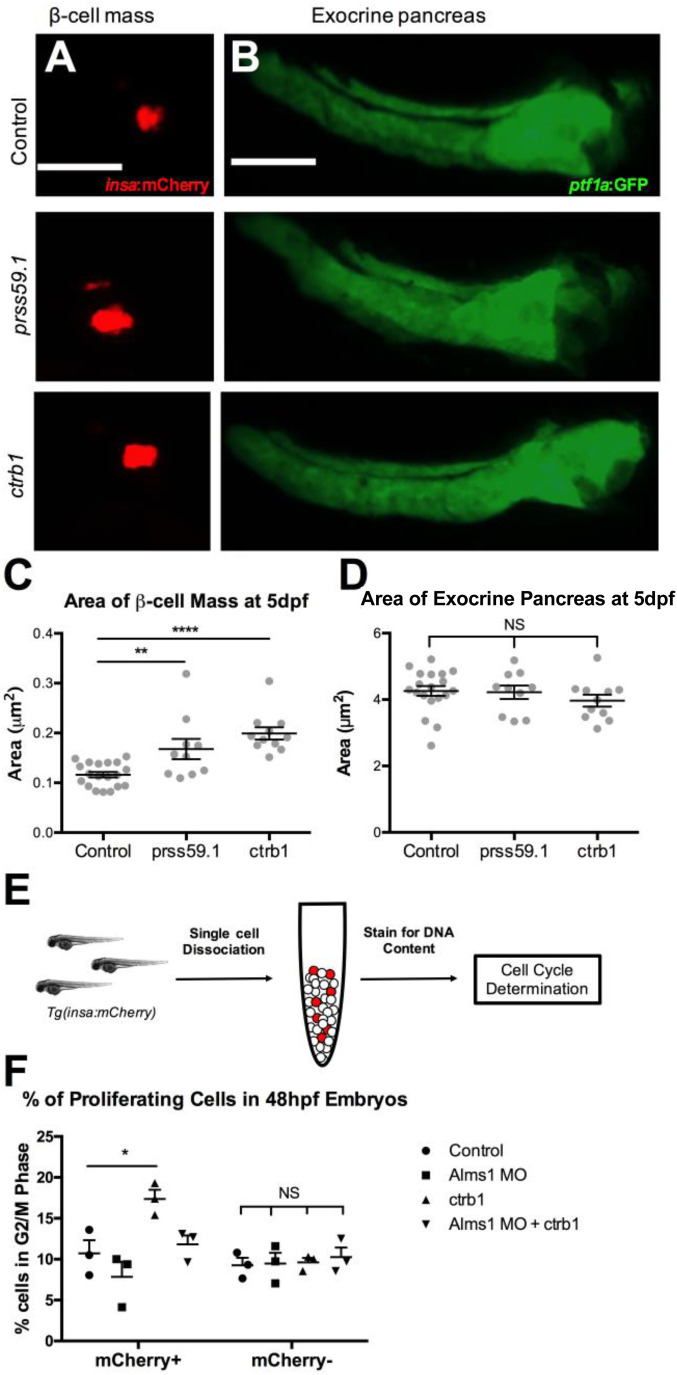
**Protease overexpression specifically increases β-cells by induction of proliferation.** (A) *insa*:mCherry expression at 5 dpf representing total area of the β-cell mass in control, *prss59.1* mRNA, and *ctrb1* mRNA injected animals. Scale bar: 100 μm. (B) *ptf1a*:GFP expression at 5 dpf representing total area of the exocrine pancreas in control, *prss59.1* mRNA, and *ctrb1* mRNA injected animals. Scale bar: 100 μm. (C) Quantification of area of mCherry fluorescence of β-cell mass in control (*n*=19), *prss59.1* mRNA (*n*=10), and *ctrb1* mRNA (*n*=11) injected animals at 5 dpf. (D) Quantification of area of GFP fluorescence of exocrine pancreas in control (*n*=19), *prss59.1* mRNA (*n*=10), and *ctrb1* mRNA (*n*=11) injected animals at 5 dpf. (E) Schematic of experimental design for cell cycle determination of β-cells in zebrafish embryos at 48 hpf. (F) Quantification of proliferating β-cells (mCherry+) and non-β-cells (mCherry−) by percentage of cells in G2/M phase in control, *alms1* MO, *ctrb1* mRNA, and *alms1* MO plus *ctrb1* mRNA injected animals at 48 hpf (*n*=3 experiments; each experiment represents *n*=50 embryos). All statistics, Ordinary one-way ANOVA, error bars represent standard error of the mean, symbols represent the following significance: NS=*P*>0.05, *=*P*<0.05, **=*P*<0.01, ****=*P*<0.0001.

Given the whole-body nature of the *ctrb1* injection rescue, we questioned whether the increase in β-cells after overexpression was β-cell specific or a result of broader changes in the whole pancreas. To test this, we utilized double transgenic zebrafish in which β-cells express mCherry driven by the *insulin* promoter and the exocrine pancreas expresses GFP driven by the *ptf1a* promoter (Pisharath et al., 2007; Wang et al., 2015). The full-length mRNAs for *prss59.1* and *ctrb1* were injected into 1–2 cell stage Tg(*insa*:mCherry),Tg(*ptf1a*:GFP) zebrafish embryos and the area of the β-cell mass and area of the exocrine pancreas were then quantified at 5 dpf (Fig. 2A,B). Consistent with the observed increase in β-cell numbers, we found a significant increase in the β-cell area in each condition: 0.17 µm^2^±0.02 for *prss59.1* and 0.20 µm^2^±0.01 for *ctrb1*, compared to control, 0.13 µm^2^±0.01 (Fig. 2C). We found no change in exocrine pancreas area after overexpression of either protease, suggesting that the effects were specific to β-cells (Fig. 2D).

Our previous observations in the ciliopathy models indicated that β-cell proliferation was a major factor in the changes in β-cell number (Lodh et al., 2015), therefore we assessed β-cell proliferation after protease overexpression. We injected either the *alms1* MO alone, *ctrb1* mRNA alone, or a combination of the *alms1* MO plus *ctrb1* mRNA into Tg(*insa*:mCherry) zebrafish embryos and dissociated the embryos into single-cell suspensions at 2 dpf. DNA was stained using Vybrant DyeCycle Violet Stain (Fig. 2E) and the cell cycle stage was assessed via DNA content, as per the manufacturer’s protocol (Fig. S3A–D). Under control conditions, we found 10.7% of β-cells to be in the G2/M phase of the cell cycle, indicating proliferating cells, and a reduction in proliferation in the AS model (7.8% in G2/M) (Fig. 2F). In contrast, there was a significant increase in β-cell proliferation upon overexpression of *ctrb1* (17.4% in G2/M, *P*=0.0122) and *ctrb1* overexpression in the AS model returned β-cell proliferation to control levels (11.8% in G2/M) (Fig. 2F). We examined the effect of each of these conditions on the non-β-cell populations by analyzing the mCherry negative cell population and found no changes in proliferation, indicating that this effect is specific to β-cells (Fig. 2F). The data suggest the increase in β-cell numbers observed upon protease overexpression is a result of increased proliferation.

### Exocrine pancreas protease proteins directly interact with β-cells *in vitro* and *in vivo*

**Fig. 3.**
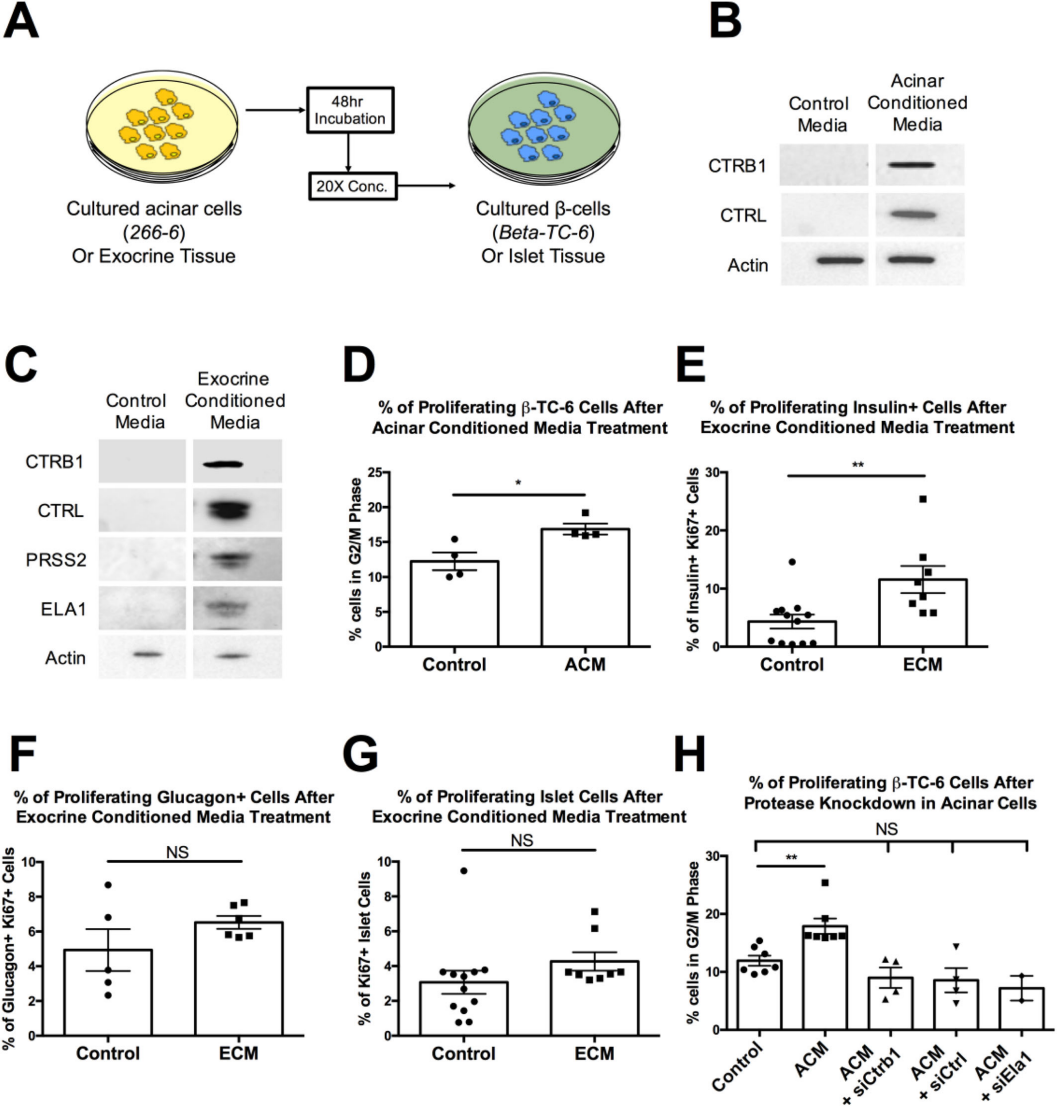
**Exocrine pancreas proteases directly interact with β-cells.** (A) Schematic of experimental design for assessing protease-β-cell interaction in cultured β-cells and islets. (B) Western blot of β-TC-6 lysate from cells treated with control media or acinar conditioned media (ACM) using anti-CTRB1 and anti-CTRL antibodies. (C) Western blot of islet tissue lysate from islets treated with control media or exocrine conditioned media (ECM) using anti-CTRB1, anti-CTRL, anti-PRSS2 and anti-ELA1 antibodies. (D) Quantification of proliferating β-TC-6 cells by percentage of cells in G2/M phase in control media or ACM treated cells. (E) Quantification of proliferating β-cells (insulin+) by percentage of Ki67 positive cells in control media or ECM treated islets. (F) Quantification of proliferating α-cells (glucagon+) by percentage of Ki67 positive cells in control media or ECM treated islets. (G) Quantification of proliferating islet cells by percentage of Ki67 positive cells in control media or ECM treated islets. (H) Quantification of proliferating β-TC-6 cells by percentage of cells in G2/M phase in control media, ACM, ACM lacking CTRB1 (ACM+siCtrb1), ACM lacking CTRL (ACM+siCtrl) and ACM lacking ELA1 (ACM+siEla1) treated cells. (**=*P*<0.01, Ordinary one-way ANOVA). Error bars represent standard error of the mean, symbols represent the following significance: NS=*P*>0.05, *=*P*<0.05, **=*P*<0.01, ***=*P*<0.001, Student's *t*-test unless otherwise indicated.

Our zebrafish findings implicate exocrine pancreas proteases in β-cell proliferation. To determine if this is a direct effect, we turned to a murine acinar cell line, a murine β-cell line, and freshly harvested mouse pancreata. Murine acinar cells (266-6, ATCC) grown in culture secrete proteases into the media, as was confirmed by western blot analysis of the culture media after 48 h of culture (Fig. S4A). This acinar-conditioned media (ACM) was concentrated and used as the protease source for cultured murine β-cells (β-TC-6) (Fig. 3A). We also isolated and separately cultured exocrine pancreas tissue and pancreatic islets from 10-week-old mice. Exocrine pancreas tissue secretes proteases into the culture media after a period of 24 h (Fig. S4E). Concentrated exocrine conditioned media (ECM) was then applied to isolated mouse islet culture (Fig. 3A). Using these systems, we first determined if β-cells take up the secreted proteases. Using western blotting and antibodies specific to mouse CTRB1 (Chymotrypsinogen B1) or CTRL (Chymotrypsin-like), we were only able to detect the proteases in cultured β-cell lysates treated with ACM (Fig. 3B). Similarly, we detected CTRB1, CTRL, PRSS2 and ELA1 in islets treated with ECM, but not those cultured in control media for the same period of time (Fig. 3C).

We examined proliferation by flow cytometry analyses in cultured cells using propidium iodide DNA staining in cultured β-cells and in β-cells of insulin+ cells from isolated islets using Ki67 (Fig. S5). In both cases we observed significant increases in proliferation. ACM treatment of cultured β-cells resulted in a 1.4-fold increase in proliferation compared to control media treated β-cells (Fig. 3D). We also found a significant, 2.7-fold increase in proliferation of insulin+ cells in *ex vivo* islets upon treatment with ECM compared to control media treated islets (Fig. 3E). Importantly, there was no significant change in proliferation when α-cells (Fig. 3F) or when all islet cells were examined (Fig. 3G), suggesting β-cell specificity for the proliferative effect. To determine if the increase in cultured β-cells was due to acinar-secreted proteases in the media, we repeated proliferation analysis after siRNA transfection targeting the proteases in the cultured acinar cells prior to media conditioning (Fig. S4A–D). While ACM from control-transfected acinar cells increased β-cell proliferation as previously, ACM from protease-depleted acinar cells lost the proliferative effect (siCtrb1 0.75-fold, siCtrl 0.72-fold, and siELA1 0.6-fold versus control ACM; Fig. 3H), suggesting the proliferation increase is specific to the proteases in the media.

**Fig. 4.**
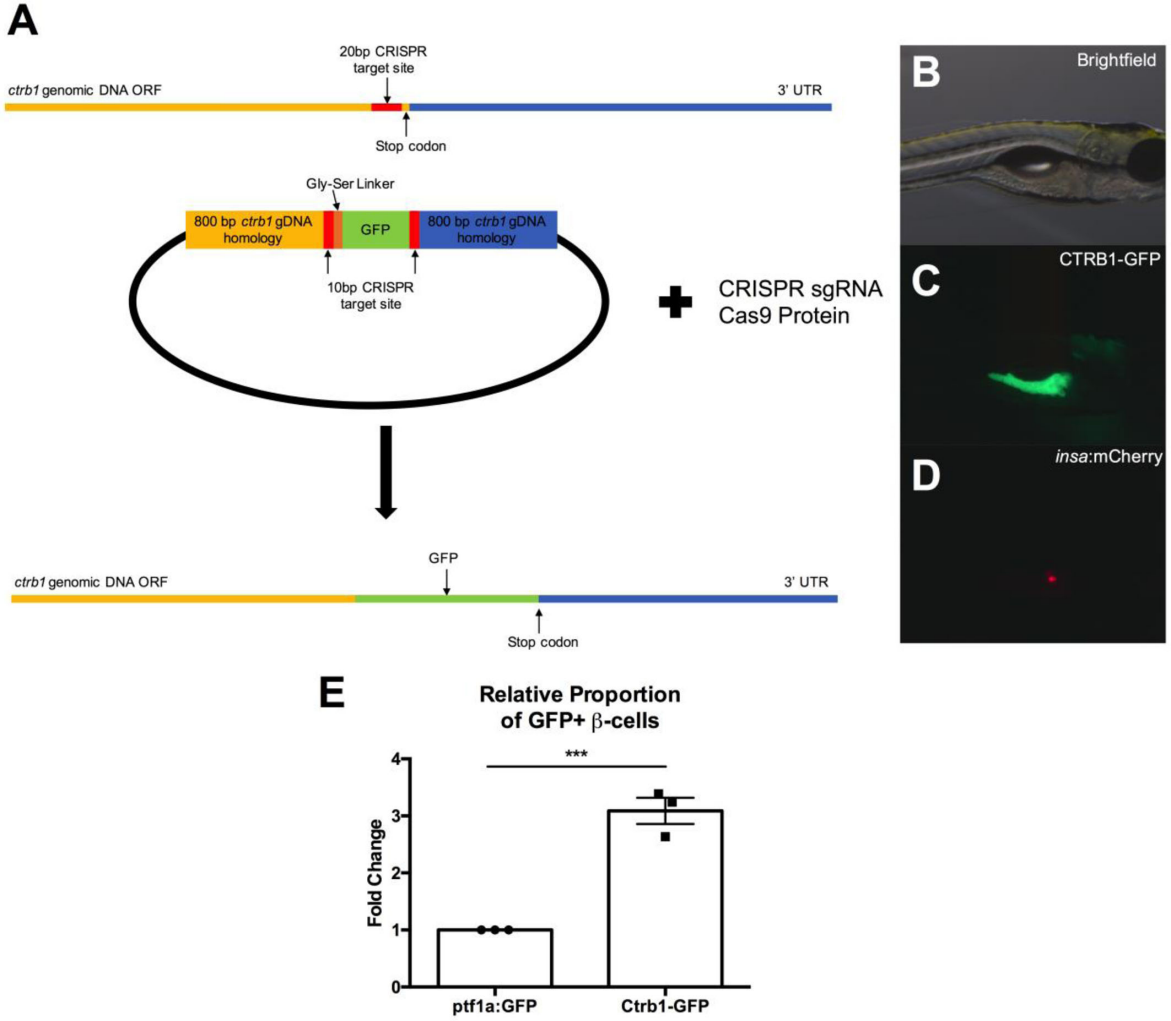
**Endogenous chymotrypsinogen B1** **in β-cells.** (A) Schematic of experimental design for generation of Tg(CTRB1-GFP),Tg(*insa*:mCherry) using CRISPR/Cas9 and homology directed repair for insertion of GFP at C-terminus of endogenous *ctrb1* gene. (B) Brightfield image of Tg(CTRB1-GFP),Tg(*insa*:mCherry) animal at 5 dpf. (C) GFP fluorescence in Tg(CTRB1-GFP), Tg(*insa*:mCherry) animal at 5 dpf indicating successful fluorescence in Tg(CTRB1-GFP),Tg(*insa*:mCherry) animal at 5 dpf indicating successful fluorescence in Tg(CTRB1-GFP),Tg(*insa*:mCherry) animal at 5 dpf. (E) Quantification of colocalization of CTRB1 in zebrafish β-cells at 5 dpf by fold change in proportion of GFP+ mCherry+ cells in Tg(Ctrb1-GFP),Tg(*insa*:mCherry) compared with Tg(*ptf1a*:GFP),Tg(*insa*:mCherry). Error bars represent s.e.m. ***P<0.001, Student's *t*-test.

**Fig. 5.**
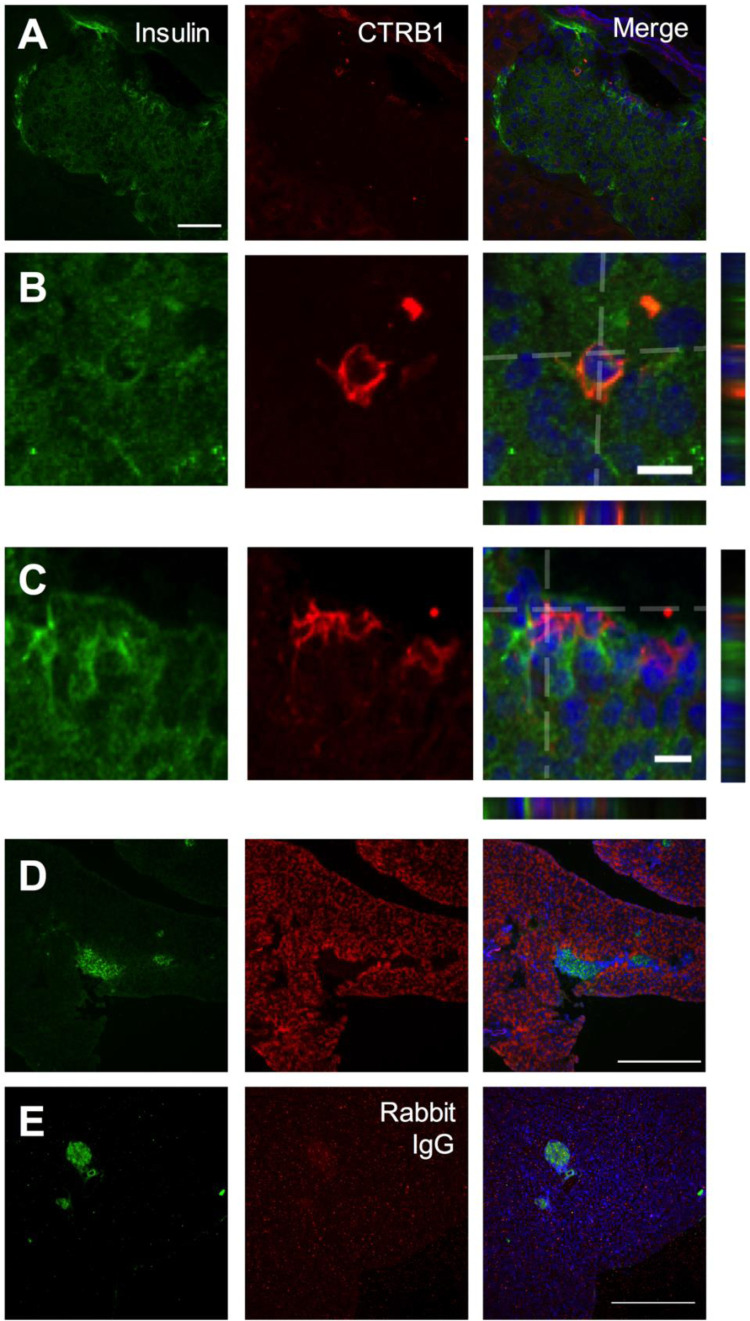
**Cryosections of mouse pancreas incubated with antibodies specific for insulin (green) and CTRB1 (red), including nuclei stained with DAPI (blue).** (A) High magnification islet view. Scale bar: 50 μm. (B,C) High magnification β-cell view. Merged image is a compressed Z-stack, orthogonal XZ and YZ are provided. Scale bars: 10 μm. (D,E) Low magnification pancreas stack, orthogonal XZ and YZ are provided. Scale bars: 10 μm. (D,E) Low magnification pancreas.

To test if our *in vitro* and *ex vivo* system observations reflect endogenous interactions *in vivo*, we returned to the zebrafish. To determine if proteases produced by the exocrine pancreas directly interact with the β-cells of the endocrine pancreas endogenously, we generated a transgenic line encoding a GFP tag at the C-terminal end of Ctrb1 in the Tg(*insa*:mCherry) line using CRISPR/Cas9 and homology-driven repair. The resulting line, Tg(Ctrb1-GFP),Tg(*insa*:mCherry), expressed GFP-tagged endogenous CTRB1 in the exocrine pancreas allowing us to visualize the tagged protein as well as β-cells within the same animal (Fig. 4A–D). We assessed colocalization of GFP and mCherry in isolated cells using flow cytometry (Fig. S6A–D). Single cell dissociations of Tg(*insa*:mCherry), Tg(*ptf1a*:GFP) zebrafish larvae, in which no colocalization is expected between the GFP and mCherry in β-cells. Compared to the *ptf1a*:GFP control we found a significant, 3.1-fold increase in the relative proportion of GFP+mCherry+ β-cells in the Tg(Ctrb1-GFP),Tg(*insa*:mCherry) transgenic line (Fig. 4E), indicating the presence of CTRB1 within β-cells. To determine if this interaction could also be found in mammalian pancreas, we performed immunofluorescent staining on mouse pancreas sections isolated from 9-week-old mice using antibodies against murine CTRB1 and Insulin (Fig. 5). In these sections we identified CTRB1 protein within a small number of insulin+ cells in three of four mouse pancreata examined (Fig. 5A–C), supporting a direct interaction between the secreted CTRB1 protease and β-cells in pancreatic islets.

## DISCUSSION

The potential for therapeutic applications in diabetes makes identification of novel approaches to increase β-cell number an active area of investigation. Discovery of endogenous regulators of β-cell proliferation can offer important insight into pathways that increase β-cell mass. Here, we report the identification of a novel interaction between pancreatic proteases (secreted from acinar cells in the exocrine pancreas) and β-cells, in the endocrine islets. Our data suggest a conserved interaction between zebrafish and mouse in which pancreatic proteases play a significant role in β-cell proliferation through direct interactions with β-cells. Together, these observations suggest a previously unappreciated role of directional crosstalk from the exocrine to endocrine pancreas that serves to regulate β-cell mass.

The vertebrate pancreas consists of separate but closely intertwined exocrine and endocrine compartments. Although both arise from common buds during development and are in very close proximity, they have traditionally been thought to be separate and non-interacting. Some lines of evidence challenge this assumption including an islet-acinar axis by which endocrine activity can influence exocrine function (Barreto et al., 2010). The reverse directionality, exocrine to endocrine, remains unclear. Recent studies have begun to shed light onto this possibility with the discovery of exocrine cell types that can contribute β-cell progenitor cells (Aguayo-Mazzucato and Bonner-Weir, 2018), or undergo β-cell trans-differentiation (Demcollari et al., 2017). Much less is known, however, about possible direct signaling interactions between exocrine cells and islets. The identification of the Reg proteins (Watanabe et al., 1994) and islet neogenesis-associated protein (Rosenberg et al., 2004) hints at exocrine-derived proteins directly stimulating β-cell neogenesis and proliferation. But very little is known about a possible signaling role for the most abundant exocrine protein class, the digestive enzyme precursor proteins. Support for this possibility has emerged recently, including genetic association data identifying variants in the gene encoding CTRB1 as risk alleles for type 1 and type 2 diabetes (‘t Hart et al., 2013; Barrett et al., 2009; Morris et al., 2012). Consistent with this, normally detectable circulating levels of pancreatic enzymes are significantly lower in type 2 diabetes patients compared to controls (Yadav et al., 2013). In addition, evidence from pig models demonstrate a role of circulating pancreatic enzymes in systemic glucose homeostasis, independent of gut digestion, which is potentially partly mediated by islet function (Lozinska et al., 2016; Pierzynowski et al., 2018). Our observations implicate individual protease proteins as potentially contributing to this phenomenon, though further experiments will be important to determine if they play compensatory roles or if each enzyme precursor protein plays a specific and distinct role. Recent reports also suggest a novel role for the serpin family of elastase inhibitors in β-cell proliferation (El Ouaamari et al., 2016; Kryvalap et al., 2016). Serpins (serine protease inhibitors) directly bind to and inhibit activated proteases, including exocrine pancreatic enzymes. Our data complement this growing body of evidence and offer support for a direct role for exocrine pancreatic enzymes in β-cell proliferation. It remains to be seen how this interaction occurs. For example, whether proteases secreted from acinar cells enter circulation or interact with β-cells locally within the pancreas is unclear. It is well established that excessive production and aberrant activation of pancreatic proteases results in pancreatitis and damages islet tissue, so it is unlikely that activated proteases would have a beneficial effect on β-cell mass. However, little is known about a possible role for the inactive zymogens. Our data suggest that inactive or inactivatable forms of at least CTRB1 can increase β-cell proliferation, providing evidence that the inactive protein may act as a signaling molecule.

Such a role would potentially be mediated by receptors on β-cells that could respond to a proliferative signal. Given that we identified a role for exocrine proteases and β-cell proliferation in models of the ciliopathies, a role for cilia cannot be ruled out. Further exploration will be needed to examine the potential involvement of cilia-regulated pathways, which may factor in to the observed proliferation phenotypes. The data presented here offer insight into an unreported mechanism by which β-cell proliferation may be mediated by endogenous factors. Together, these observations inform our understanding of pancreas biology and potentially assign a new signaling role to exocrine proteases in regulating endocrine β-cells.

## MATERIALS AND METHODS

### Zebrafish lines

Experiments were carried out using the following zebrafish lines: Tubingen (WT), Tg(*insa*:mCherry), which labels β-cells specifically by expressing mCherry under the control of the *preproinsulin* (*insa*) promoter, and Tg(*ptf1a*:GFP),Tg(*insa*:mCherry), which expresses GFP under the control of exocrine pancreas marker *ptf1a* along with mCherry under the control of the *insa* promoter (Pisharath et al., 2007). Adult zebrafish of both sexes were housed and naturally mated according to standard protocol. All zebrafish work was conducted in accordance with University of Maryland IACUC guidelines.

### Cloning

Protease coding sequences were amplified from cDNA generated from RNA isolated from WT zebrafish using the following primers: *prss59.1*, forward: 5′CATGGAATTCATGAGGTCTTTGGTGTTCCTGG, reverse: 5′CGTCTAGATTAGTTGTTTCTCATGGTGTCGG; *cela1*, forward: 5′CATGGAATTCCTGCAACATGCTGAGGATCCTG, reverse: 5′CGTCTAGAGATTGTTCAGCTTATTTAGCAAT; *ctrb1*, forward: 5′CATGGAATTCATTCAACTGCAGCAATGGCC, reverse: 5′CGTCTAGATTAGTTGGAAGCAATGGTCTGG; *try*, forward: 5′CATGGAATTCATGAAGGCTTTCATTCTTCTGGC, reverse: 5′CGTCTAGATCATGGTGTTTCTGATCCAGG.

Amplified fragments were cloned into pCS2+, grown with ampicillin resistance (Sigma-Aldrich), and sequence confirmed.

### Site directed mutagenesis

Inactivatable *ctrb1* was generated by mutating the isoleucine at the cleavage site to an alanine and catalytically dead *ctrb1* was generated by mutating the histidine of the catalytic triad to a tryptophan using the Q5 Site-Directed Mutagenesis Kit (NEB) and the following primers: Inactivatable, forward: 5′CTACGCCAGGGCGGTGAATGGTGAGG, reverse: 5′CCGGTAACAACTGGAGGG; Catalytically Dead, forward: 5′GACTGCTGCTTGGTGCAACGTTAG, reverse: 5′ACAACCCAATTCTCATTG.

Mutated plasmids were grown with ampicillin resistance and sequence confirmed.

### *In vitro* transcription and mRNA injection

Protease mRNA was transcribed using mMessage mMachine kits (Thermo Fisher Scientific). 50 pg, 100 pg, 150 pg, 200 pg and 250 pg of each protease mRNA was injected into one- to two-cell stage embryos to determine optimal concentrations, after which the following amounts were used: 100 pg *prss59.1*, 100 pg *cela1*, 200 pg *ctrb1* and 150 pg *try*. The embryos were grown at 28°C until harvesting for analyses.

### Morpholinos

MOs that block splicing (SB) of targeted mRNAs were injected into one- to two-cell stage embryos. We used previously validated MOs to target *alms1* and *bbs1* (Leitch et al., 2014; Lodh et al., 2015) and designed SB MO (5′-ATAAAGCCTGTCACTCACTGGAGCT-3′) to target *prss59.1* transcript and SB MO (5′-ACAGATTTTAGACTGTACGCACCTT-3′) to target *ctrb1* transcript. A control non-specific MO was used (5′-CCTCTTACCTCAGTTACAATTTATA-3′).

### β-cell and exocrine pancreas analysis

β-cell number, mass area and exocrine pancreas area were determined as previously described (Lodh et al., 2015). Briefly, the size of the β-cell mass (area of mCherry expression) and the size of the exocrine pancreas (area of GFP expression) were quantified using a Zeiss Lumar v12 stereomicroscope and ImageJ software. The number of β-cells was quantified by fixing the embryos at 5 days post fertilization (dpf) in 4% paraformaldehyde (PFA), dehydrating in 100% methanol, and flat mounting in ProLong Gold antifade (Thermo Fisher Scientific) with the right lateral side facing the coverslip. Sufficient pressure was applied to disrupt the islets in order to visualize individual cells. The number of β-cells was counted manually under an Olympus ix50 with cellSense imaging software at 20X magnification.

### Cell culture

β-TC-6 cells (CRL11506; ATCC) were cultured in DMEM-H (ATCC) supplemented with 15% heat-inactivated FBS and 1X penicillin/streptomycin (Sigma-Aldrich). 266-6 (CRL2151; ATCC) were cultured in DMEM supplemented with 10% heat inactivated FBS and 1X penicillin/streptomycin. Knockdowns were accomplished using Lipofectamine 3000 (Thermo Fisher Scientific) and either scrambled control siRNA or siRNAs targeting *alms1*, *bbs1*, *ctrb1*, *ctrl1*, or *ela1* (Thermo Fisher Scientific). Efficacy of siRNA knockdown was evaluated via qRT-PCR.

Acinar conditioned media (ACM) was collected after 48 h incubation on 266-6 cells and concentrated 20-fold by centrifugation using 10 K Macrosep Advance Centrifugal Devices (Pall). 500 uL of concentrated ACM was applied to 10 million Beta-TC-6 cells for 8 h before harvesting for analyses.

### Mouse islet isolation and culture with exocrine conditioned media

All experiments were performed in accordance with University of Maryland IACUC guidelines.

Islets were isolated from 10-week-old C57BL/6 male mice (kindly gifted from the Bromberg group through the University of Maryland Animal Resources) following established protocols (Ashari et al., 2018; Szot et al., 2007), picked, and plated in supplemented RPMI medium 1640 [10% fetal bovine serum (FBS; Gemini), 1% penicillin–streptomycin (P/S) (Invitrogen)]. Following Ficoll separation from the islets, the exocrine tissue was washed twice in PBS (Corning) containing 2.5 mM EDTA (Corning), plated in supplemented RPMI+1% P/S, and cultured for 24 h at 37°C before harvest of the media supernatant. The media supernatant was then concentrated 10:1 using a centrifugal concentrator (Macrosep Advance Centrifugal Filter; Pall Laboratories) and applied to the islet cells in culture for 5 days.

### Flow cytometry and DNA stain

Pooled groups of 50 zebrafish embryos were dissociated to single-cell suspensions following published protocols (Samsa et al., 2016) using TrypLE (Thermo Fisher Scientific) followed by FACSMAX (Genlantis) until a single-cell dissociation was achieved. Dissociated cells were suspended in Hank's Balanced Salt Solution (HBSS) supplemented with 10% Fetal Bovine Serum (FBS) and kept on ice. DNA staining was done using Vybrant DyeCycle Violet Stain (Thermo Fisher Scientific) according to the manufacturer's protocol. Samples were analyzed on a BD LSR II flow cytometer.

Cell samples were collected, washed in PBS, fixed in 70% ice-cold ethanol for 10 min, and suspended in FACS buffer in a single cell suspension. DNA staining was performed using Propidium Iodide (Thermo Fisher Scientific) according to the manufacturer's protocol. Samples were analyzed on a BD Canto II flow cytometer.

Islets were dissociated (Cell Dissociation Buffer, enzyme-free, Hanks' Balanced Salt Solution; Gibco) at 37°C for 10 min and washed in PBS containing 2.5 mM EDTA (Corning). Flow cytometry was performed as previously described (Ashari et al., 2018) using isotype- and fluorophore-matched FACS antibody staining with Alexa Fluor 647 IgG1 mouse anti-insulin (BD Pharmingen), Pacific Blue IgG2a mouse anti-glucagon pacific Blue (R&D Systems) and PE Cy7 IgG2a rat anti-Ki67 (BioLegend). The positive gates for each parameter were established using single-stained isotype controls, Alexa Fluor 647 mouse IgG1 (BioLegend) and PE Cy7 rat IgG2a (eBioscience) with respective positive thresholds for insulin, glucagon, and Ki67 including 1% of the cells in the corresponding isotype-stained sample. In experimental samples, events with a fluorescence intensity exceeding this positive threshold were defined as being positive for the respective parameter.

### Western blots

Cells were harvested in Tris-Triton buffer with protease inhibitors (Sigma-Aldrich), run via SDS-PAGE and blotted onto polyvinylidene fluoride (PVDF) membranes. Membranes were incubated overnight with rb- αCTRB1 (1:3000; Proteintech), rb-αCTRL (1:1000; Proteintech), rb-αPRSS2 (1:400; Proteintech), rb-αELA1 (1:500; Sigma-Aldrich), or rb-αActin (1:5000; Sigma-Aldrich), then 1 h at room temperature with species specific HRP-conjugated secondary antibodies (goat anti-rb 1:30,000; Jackson ImmunoResearch), and exposed with SuperSignal West Femto Maximum Sensitivity Substrate (Thermo Fisher Scientific). Protein intensity was normalized to actin and quantified via densitometry function in ImageJ.

### Transgenic line generation

The endogenous GFP -tagged CTRB1 was generated using the CRISPR/Cas9 system and homology directed repair. A CRISPR target site closest to the stop codon within the ORF (5′-GGGTGACTCTGGTGGTCCTC-3′) was identified. Creation of the oligo included incorporation of a T7 promoter ahead of the target sequence (TAATACGACTCACTATA) and an overlap sequence after (GTTTTAGAGCTAGAAATAGC) as previously described (Lodh et al., 2015; Varshney et al., 2015). The sgRNA was generated by annealing this oligo to a second, common, oligo (5′-AAAAGCACCGACTCGGTGCCACTTTTTCAAGTTGATAACGGACTAGCCTTATTTTAACTTGCTATTTCTAGCTCTAAAAC-3′). The donor sequence was designed as a gblock to contain an 800 bp homology arm ahead of the CRISPR target site followed by the first 10 bp of the target site, a glycine-serine linker, the GFP coding sequence, a stop codon, the remaining 10 bp of the CRISPR target site, and an 800 bp homology arm.

The gblock was cloned into a pCR Blunt II-TOPO vector using the Zero Blunt TOPO PCR Cloning Kit (Thermo Fisher Scientific) with kanamycin resistance (Sigma-Aldrich). The sgRNA, Cas9 protein, and donor plasmid were microinjected directly into the during the single-cell stage of embryonic development. Specific integration of the GFP sequence was verified by PCR amplification and sequencing of genomic DNA extracted from transgenic animals using primers flanking the region as well as primers within the GFP sequence to ensure integration at only the target site.

### Immunofluorescence

Mouse pancreata were snap frozen in OCT and cryosections were cut 10 µm thick, then fixed in 100% cold methanol. Sections were then incubated overnight with gp-αINS (1:50; Abcam) and rb-αCTRB1 (1:100; Proteintech), then 1 h at room temperature with species specific fluorescence-conjugated secondary antibodies (goat anti-gp 488 1:200 and goat anti-rb 594 1:200; Thermo Fisher Scientific), and mounted with ProLong Gold antifade (Thermo Fisher Scientific).

### qRT-PCR

RNA was extracted using RiboZOL RNA Extraction Reagent (VWR) and converted to cDNA via First Strand cDNA Synthesis (Thermo Fisher Scientific) per manufacturer protocols. Gene expression was determined on a LightCycler 480 (Roche) using 2X SYBR Green Master Mix (Roche) and compared by ΔΔCT. Actin and GADPH were used as controls in zebrafish and cultured cells, respectively. Primer sequences are available upon request.

### Statistical analyses

All experiments represent a minimum of three replicates, with sample number (*n*) provided. Prismv6.0 (GraphPad) was used to determine appropriate analyses and statistical significance (indicated in figure legends).

## Supplementary Material

Supplementary information
