# Supplementary material for: Exocrine pancreas proteases regulate β-cell proliferation in zebrafish ciliopathy models and in murine systems
Source: Biol Open. 2021 Jun 14;10(6):bio046839. doi: 10.1242/bio.046839 (PMC8249909; doi:10.1242/bio.046839)
Supplement: Supplementary information [file biolopen-10-046839-s1.pdf]

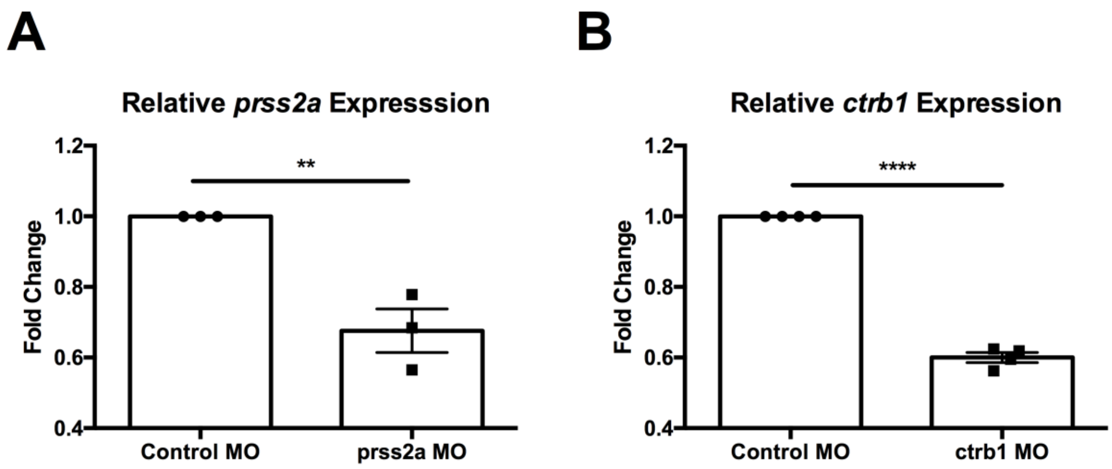

Figure S1. Morpholino (MO) knockdown of protease expression. **(A)** Quantification of fold change in *prss59.1* expression in control and *prss59.1* MO injected zebrafish at 5dpf. **(B)** Quantification of fold change in *ctrb1* expression in control and *ctrb1* MO injected zebrafish at 5dpf. All statistics, Student's t-test, error bars, SEM, **p<0.01, ***p<0.001.

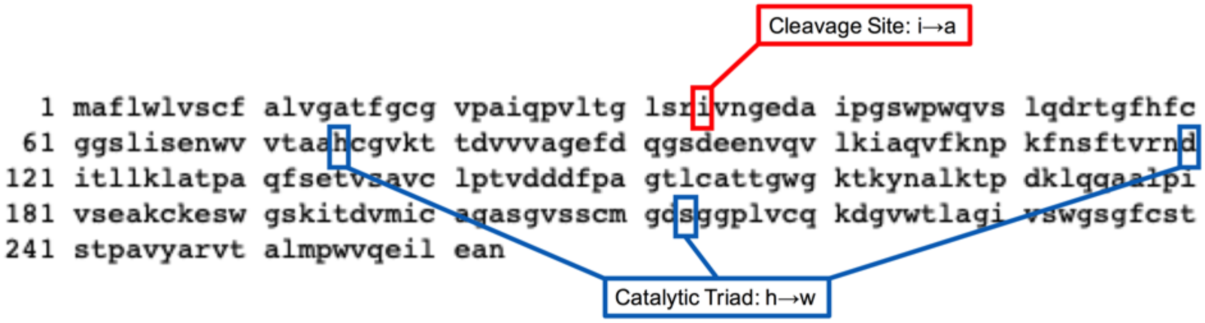

Figure S2. Generation of inactivatable and/or catalytically dead *ctrb1* mutants. Amino acid sequence of CTRB1 showing the activating cleavage site (red), wherein the isoleucine is changed to an alanine to prevent activation by cleavage, and the catalytic triad (blue), wherein the histidine is changed to a tryptophan to inhibit catalytic activity while maintaining protein structure.

Biology Open • Supplementary information

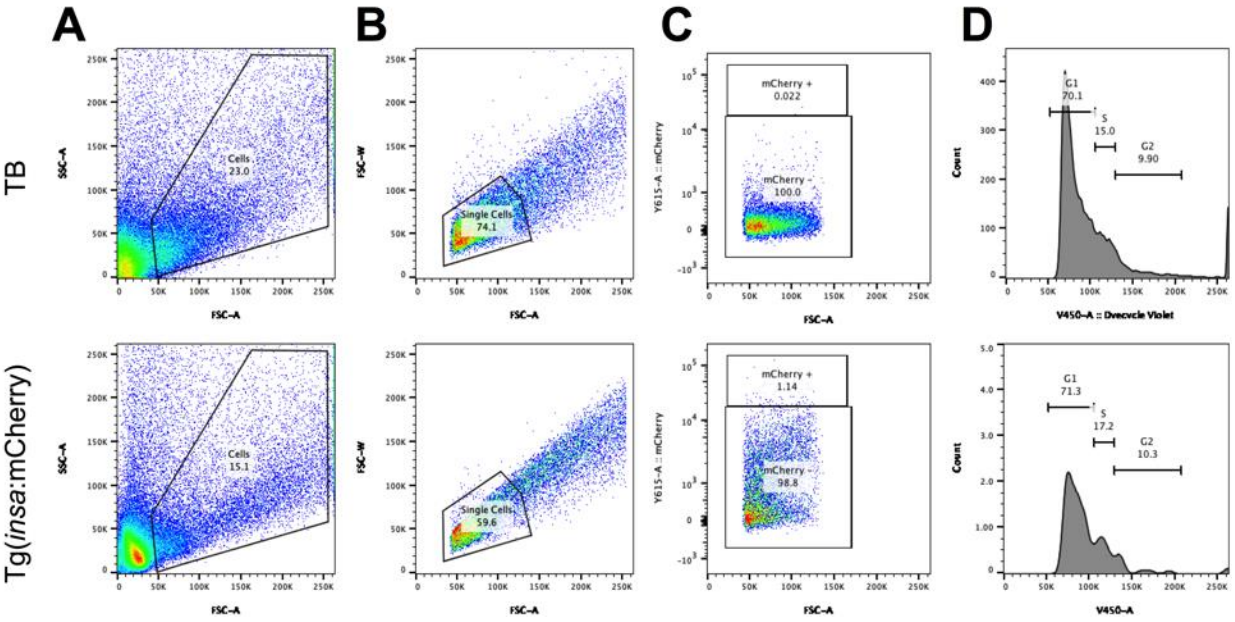

Figure S3. Gating strategy for cell cycle determination of zebrafish cells in Tubingen (top) and Tg(*insa*:mCherry) (bottom) animals. **(A)** Gating strategy for cells. **(B)** Gating strategy for single cells. **(C)** Gating strategy for mCherry+ (β) and mCherry- (non-β) cells. **(D)** Gating strategy for G1, S, and G2/M phases of the cell cycle.

Biology Open • Supplementary information

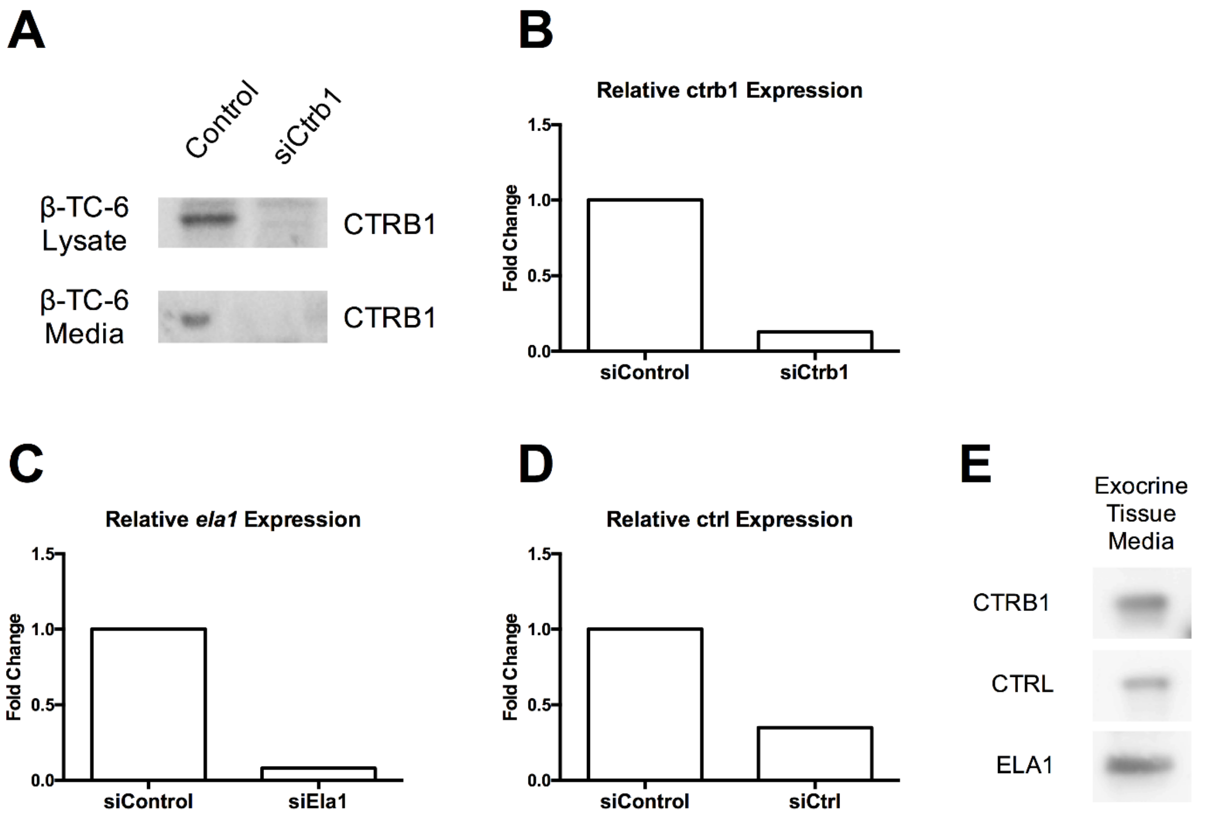

Figure S4. Protease production and secretion from acinar cells and exocrine tissue. **(A)** Western blot for CTRB1 in 266-6 cell lysates (top) or media cultured with 266-6 cells (bottom) transfected with control siRNA or siRNA targeting *ctrb1* transcripts. **(B)** Quantification of fold change in *ctrb1* expression in control and siCtrb1 transfected 266-6 cells. **(C)** Quantification of fold change in *ela1* expression in control and siEla1 transfected 266-6 cells. **(D)** Quantification of fold change in *ctrl* expression in control and siCtrl transfected 266-6 cells. **(E)** Western blot for proteases in media cultured with exocrine tissue.

Biology Open • Supplementary information

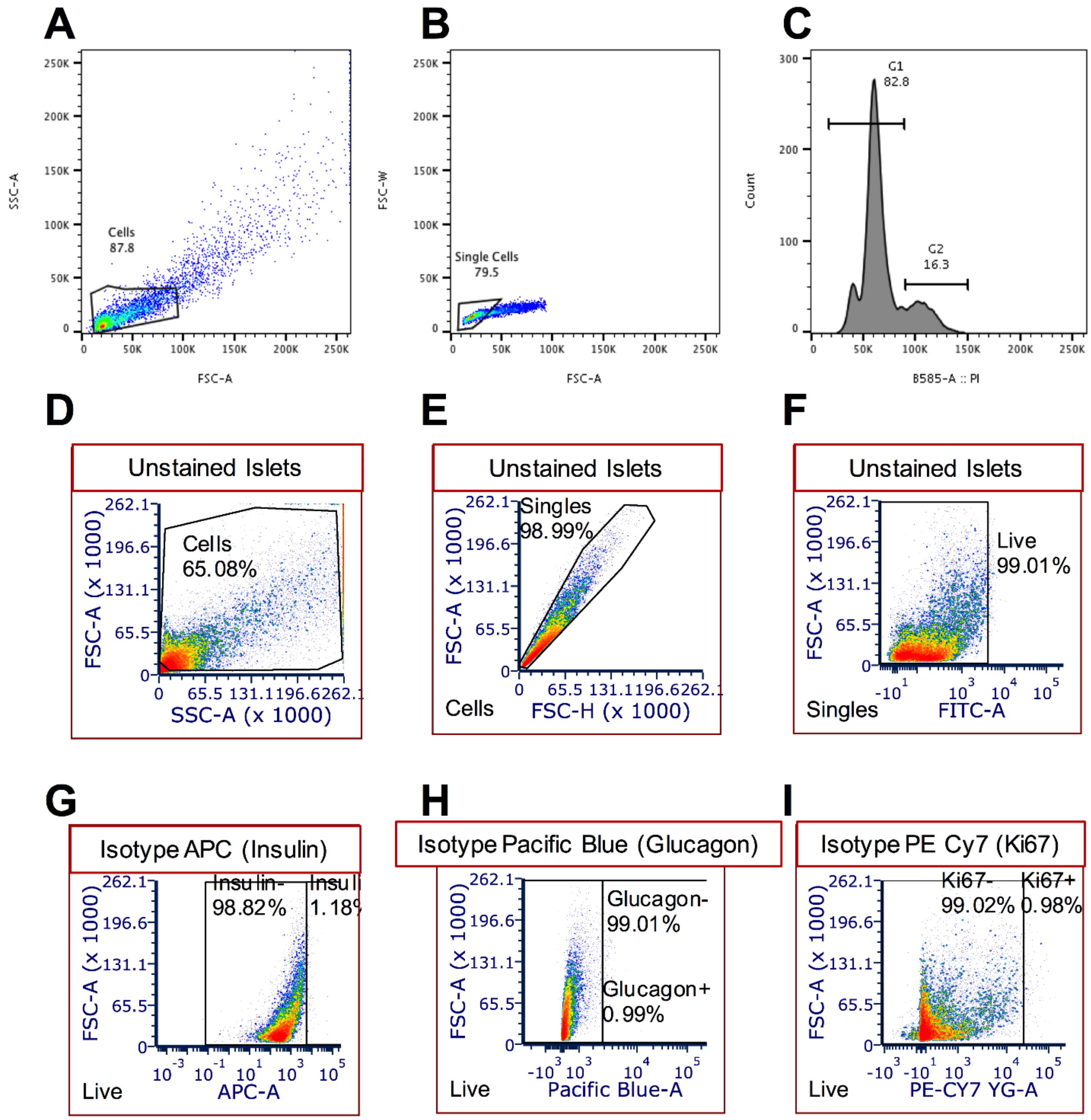

Figure S5. Gating strategy for flow cytometry of β-TC-6 cells **(A-C)** and isolated islets **(D-I)**. **(A)** Gating strategy for cells. **(B)** Gating strategy for single cells. **(C)** Gating strategy for G1 and G2/M phases of the cell cycle. **(D)** Gating strategy for cells. **(E)** Gating strategy for single cells. **(F)** Gating strategy for live cells. **(G)** Gating strategy for Insulin+/- cells. **(H)** Gating strategy for Glucagon+/- cells. **(I)** Gating strategy for Ki67+/- cells.

Biology Open • Supplementary information

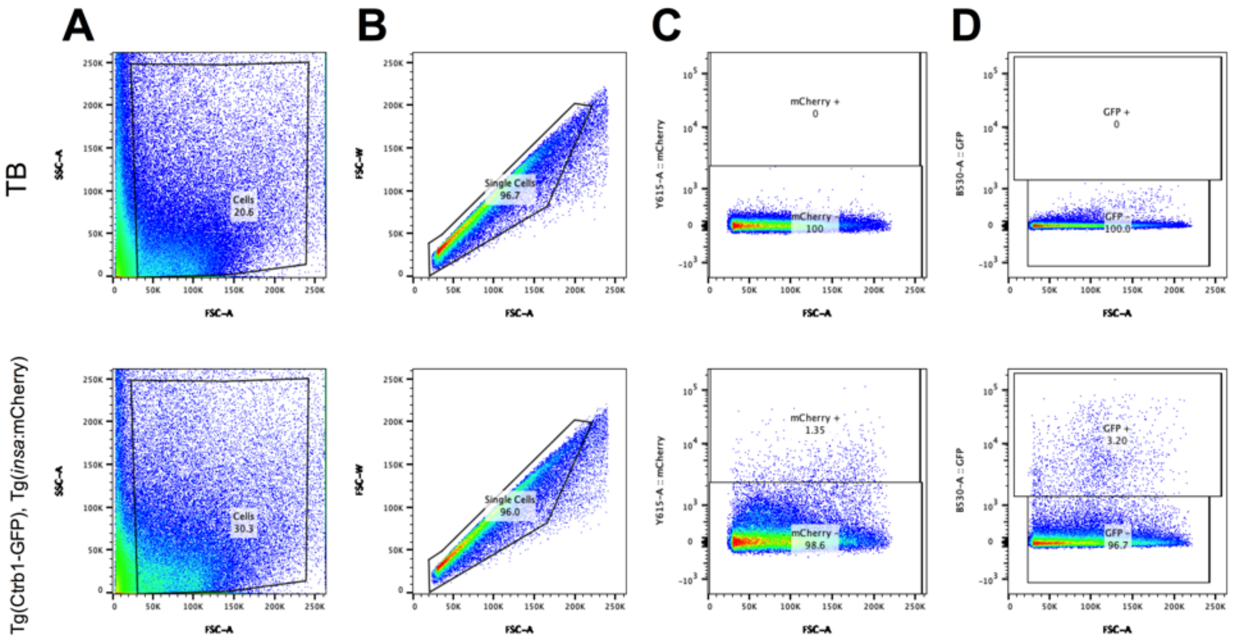

Figure S6. Gating strategy for identifying GFP+ mCherry+ cells in Tubingen (top) Tg(CTRB1-GFP),Tg(*insa*:mCherry) (bottom) animals. **(A)** Gating strategy for cells. **(B)** Gating strategy for single cells. **(C)** Gating strategy for mCherry+ and mCherry- cells. **(D)** Gating strategy for GFP+ and GFP- cells.

Biology Open • Supplementary information
